# Ki-67 and p53 correlation prognostic value in squamous cell carcinomas of the oral cavity and tongue

**DOI:** 10.1016/S1808-8694(15)30494-8

**Published:** 2015-10-19

**Authors:** Rafael Da Ros Motta, Claudio Galeano Zettler, Eduardo Cambruzzi, Geraldo Pereira Jotz, Renata Brutti Berni

**Affiliations:** 1 Pathologist; 2 Pathologist. Adjunct Professor of Medicine - ULBRA. Professor - Graduate Program in Pathology - FFFCMPA; 3 Pathologist. Adjunct Professor of Medicine - ULBRA; 4 Otorhinolaryngologist. Adjunct Professor - Department of Morphological Sciences - UFRGS. Adjunct Professor of Medicine - ULBRA; 5 Resident Physician in Pathology. Universidade Luterana Do Brasil - ULBRA Departamento de Patologia

**Keywords:** ki67 antigen, squamous cell carcinoma, oral cavity, p53, prognosis

## Abstract

Epidermoid carcinomas represent from 90% to 95% of oral cavity malignant neoplasias, making up 13,470 cases/year.

**Aims:**

To correlate p53 and Ki-67 expressions in mouth and tongue carcinomas with lymph node status, gender, histological grade, tumor volume and pathological stage.

**Materials and Methods:**

We carried out a retrospective study of 28 cases of mouth and tongue epidermoid carcinomas. They were submitted to immunohistochemical study in order to check the expression of p53 and Ki-67 antibodies and statistically compare them in terms of lymph node status, gender, histological grade, tumor volume and pathological staging.

**Results:**

The individually analyzed p53 proved to have statistical significance (p < 0.05) when compared to tumor volume (p=0.029). Despite a strong tendency, the p53/tumor volume relation was not significant. When p53 + Ki67 were analyzed, tumor volume had p < 0.05 (p = 0.029).

**Discussion:**

Literature shows that the expression of p53 and Ki-67 is related to the presence of metastasis to lymph nodes and a worse prognosis.

**Conclusion:**

In oral cavity and tongue epidermoid carcinomas, p53 and Ki-67 are related to larger tumors, metastasis to lymph nodes and very likely to a worse prognosis.

## INTRODUCTION

Epidermoid carcinomas represent 90% to 95% of the oral cavity malignant neoplasias, especially involving the posterior lateral border of the tongue. It usually affects men above 50 years of age, most with a past of high consumption of tobacco, alcohol, excessive exposure to sun light, bad mouth hygiene, diets poor in fruits and vegetables, secondary infection by the human papillomavirus (HPV) and Herpes Simplex Virus (HSV-1), and these factors are considered of high risk for cancer development^1^, ^2^, ^3^.

The tongue and oral cavity epidermoid carcinoma is responsible for approximately 30,990 new cases of cancer in the USA per year - according to 2006 estimates, of which 7,430 deaths are expected. It is responsible for 1.3% of all cancer-related deaths. In Brazil we estimate an incidence of around 13,470 new cases, 10,060 in men and 3,410 in women. Epidermoid carcinoma is the most common malignant neoplasia in the oral cavity, being responsible for 2.1% of all the malignant neoplasias in men and 0.7% in women. The age of highest incidence is the seventh decade of life, frequently associated to leaves of absence from work. The prognostic evaluation of this neoplasia is given by histological and immunohistochemical findings, including staging, histological grade, tumor size, lymph node involvement, immunohistochemical expression of p53 and Ki-67. The staging of this neoplasia is done according to the TNM system of the International Union Against Cancer (UICC), with assessment of tumor size and lymph node involvement. Epidermoid carcinomas can be differentiated or keratinized (histological grade I), or moderately or little differentiated (histological grades II and III). Invasive neoplasias which measure from 0 to 2.0cm are classified as T1 and the ones measuring 2.0 to 4.0 are T2 and the ones larger than 4cm are T3. When adjacent tissue is invaded, they are graded as stage T4, regardless of size. Even small lesions can be classified as T4, which are associated with a worse prognosis.

With the technological progress in image diagnostic methods (CT scan and MRI), the medical knowledge also broadens. Medical literature has had a growing number of papers trying to assess tumor volume as an independent prognostic factor in carcinomas that affect the tongue, nasopharynx and lungs. Some studies are able to prove this theory; however the literature is still very controversial.

Lymph node involvement is still considered one of the major prognostic markers of these neoplasias. According to the TNM system, the involvement of a single ipsilateral lymph node of up to 3cm in its largest axis is classified as N1. N2 corresponds to the cases with single metastasis in ipsilateral lymph node measuring between 3 and 6cm or multiple uni or bilateral lymph nodes smaller than 6cm. In N3 we find the cases with metastasis to lymph nodes larger than 6cm in diameter.

Immunohistochemical tests have been used in the assessment of other prognostic factors associated with oral cavity and tongue carcinomas, p53 and Ki-67 are among them. The relationship between p53 expression or that of Ki-67 cell proliferation marker with mouth epidermoid carcinoma has been broadly studied in current times. This may be due to the social impact caused by this neoplasia. Cancer suppressor genes or those that regulate apoptosis represent an important variable in the equation of neoplasias, among them p53 stands out^4^, ^5^, ^6^. Results from a large number of studies have shown that p53 expression has a major prognostic meaning in different types of cancer. P53 is a proto-oncogene and has its role involved in controlling the cell death cycle, consequently in genomic stabilization. Mutated genes become oncogenes and lose their property of removing an altered cell from the cell cycle, repair it and take it back to the cycle or lead it to programmed cell death, thus causing carcinogenesis. P53 protein expression can be easily detected in approximately 50-60% of the cases of oral cavity or tongue carcinoma, which may correspond to an increase in protein expression or even a mutation^7^, ^8^, ^9^.

Today, uncontrolled cell proliferation is considered one of the most important biological mechanisms associated with oncogenesis^10^. Results from a large number of studies show that the proliferation activity has a major prognostic meaning in a variety of tumors. Cell proliferation markers have been classified in three categories: [1] growth fraction markers; [2] markers of specific cell cycle phases; and [3] cell cycle time markers. Growth fraction that is the ratio of cells growing within the cycle can be easily identified by Ki-67 or MIB-1 antibodies, identifying antigen expression on phases G1, S, G2 and M of the cell cycle^11^,^12^.

## OBJETIVES

The goal of the present study was to check the correlation of p53 and Ki-67 immunohistochemical expression in oral cavity and tongue epidermoid carcinomas with lymph node stage (pN), as well as to observe marker expression and correlate tumor volume, patient gender, degree of histological differentiation and pathological staging (pT).

## MATERIALS AND METHODS

We carried out a cross-sectional study with twenty-eight cases of oral cavity and tongue epidermoid carcinomas, with positive regional lymph nodes for metastasis, operated upon and diagnosed between November of 1999 and April of 2006, corresponding to a period of 78 months. We reviewed all the tongue and oral cavity tumors found in the corresponding period of time, and we took off the current study all the neoplasias which were not epidermoid carcinomas. As inclusion criteria we used the existence of a sample (block) for each case, a proper block conservation status and proper histological grading used when the diagnosis was standardized by the WHO. Thus, 28 oral cavity or tongue epidermoid carcinomas with neck lymph node resection were selected. As to lymph node involvement we used as criteria the presence of metastasis seen under light microscopy, the cases were broken down into two groups: (1) cases with negative metastatic lymph nodes and (2) cases with positive metastatic lymph nodes. As to the histological grade of the neoplasia, the cases were broken down into two groups: (1) low grade (including grade I / well differentiated) and (2) high grade (including grades II and III / moderately and with little differentiation). As to pathological staging (pT) of the neoplasias, the cases were broken down into two groups: (1) lesions with local involvement (including pT1, pT2, pT3) and (2) lesions involving neighboring structures (pT4). As far as tumor volume is concerned, the neoplasias were broken down into two groups: group (1) in which the neoplasias had a volume equal to or lower than 8cm3 and group (2) in which the neoplasias had a volume greater than 8cm3. Tumor volume calculation is done by the multiplication of the three values found during the macroscopic exam of the specimen, longitudinal axis × width × depth.

Sample collection was authorized by the guardians, and the research project was approved by the Ethics in Research Committee under protocol # CEP-2006-246H, within the standards which regulate research with human beings, according to resolution # 196/96 of the National Health Council - Department of Health - Brazil.

### Immunohistochemistry

The cases were submitted to an immunohistochemistry technique to check the expression of antibodies p53 and Ki67. For the immunohistochemical technique we used sections of tissues previously fixed in 10% formalin, included in paraffin blocks. From each sample we obtained histological cross-sections of 3 micrometers of width, which were placed on silanized slides. For the test itself, each slide was sent for deparaffinization with xylol and cross-section hydration with ethanol. Antigenic recovery was carried out in two microwave ovens, with a 10mM/pH 6.0 nitric oxide solution in two 9-minute cycles each, at a power of 750W. The endogenous peroxidase block was carried out with 3% hydrogen peroxide (10 volume). The primary antibody was diluted in a 1% albumin solution in PBS, incubated in a moist chamber at 37?C, and then the material was placed under a 4?C refrigeration for 18 hours. Biotinilated antibody used a moist chamber at 37?C, for 30 minutes, just like the incubation with the streptavidin-biotin-peroxidase (StreptABC) complex. The chromogenic substrate used corresponded to a 60 mg% diaminobenzidine in PBS, as for dye we used Harrys hematoxylin. The antibodies used were the p53 protein, DO-7 clone, monoclone (DAKO Corporation) and the Ki-67 antigen, MIB-1 clone, monoclonal (DAKO Corporation). The immunohistochemical expression of each antibody for each case was estimated by the percentage of cells expressing a specific color regarding the antibody used, and the following quantification scale was used, according to the Department of Pathology's Standards, since there is no consensus in the literature about the criteria for positivity:

Negative expression: 0 to 09% of the neoplastic cells having antibody expression.

**Figure 1:**
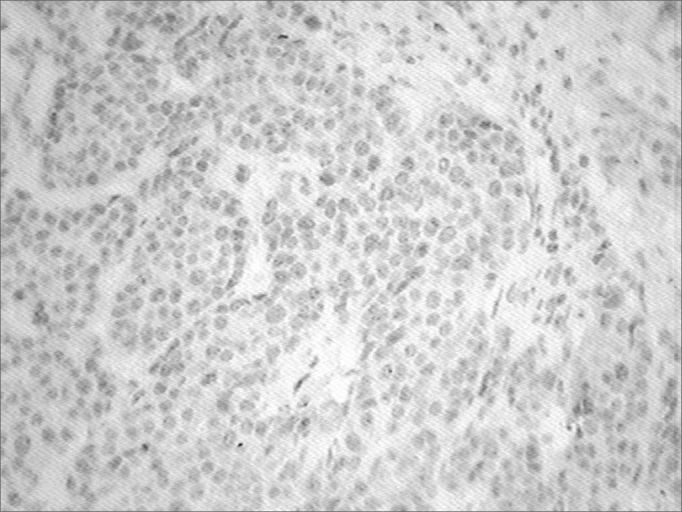
Immunohistochemistry-p53 - Cells dyed in brown show a positive reaction.

Positive expression: 10 to 100% of the neoplastic cells having antibody expression. (Figure 1)

### Statistics

The statistical analysis was carried out using the SPSS version 12.0 software starting from the chi-squared test to compare categorical variables. Through the statistical analysis we assessed the combined relation between Ki-67 and p53 tumoral expression with the other variables studied (lymph node status, gender, histological grade and pathological staging of the tumors [pT]). The values were considered statistically significant when the p value was lower than 0.05 or when necessary we used the Fisher's exact test, considered valid when f is lower than 0.05.

## RESULTS

The study included 28 patients with histological diagnosis of epidermoid carcinoma of the tongue and oral cavity submitted to surgery with neck lymph node resection.

As far as gender is concerned, 24 patients were males and 4 were females, which corresponded to 85.7% and 14.3% respectively. As to tumor differentiation, 2 were classified as being of low grade and 26 as high grade, corresponding to 7.15% and 92.85%. As to tumor staging, 10 patients had tumors which did not invade adjacent structures (pT1, 2, 3) and 18 had tumors invading adjacent structures (pT4), corresponding to 35.7% and 64.3% respectively. Lymph node status was defined as positive in 14 cases and negative for metastasis in 14 cases. As to the relationship to the expression of P53 and Ki67 immunohistochemical markers, p53 was expressed in 50% of the cases and Ki-67 expression happened in 92.85% of the cases and the simultaneous expression of both happened in 50% of the cases. In terms of tumor volume, 25% of the tumors were smaller or equal to 8cm^3^(7 cases) and 75% of the tumors were larger than 8cm^3^, corresponding to 21 cases. (Table 1)

**Table 1:** Statistical correlation of the variables studied with p53, ki-67 and p53 + Ki-67 markers

Variables studied	p53	Ki-67	p53&Ki-67
Gender	p = 0,280	p = 0,134	p = 0,280
	f = 0,595	f = 0,269	f = 0,595
pT	p = 0,114	p = 0,166	p = 0,114
	f = 0,236	f = 0,523	f = 0,236
pN	p = 0,023	p = 0,075	p = 0,023
	f = 0,056	f = 0,481	f = 0,056
Tumor volume	p = 0,029	p = 0,269	p = 0,029
	f = 0,076	f = 1,000	f = 0,076
Histological grade	p = 0,075	p = 0,578	p = 0,075
	f = 0,481	f = 0,231	f = 0,481

Values < 5 in two Chi2 squares can not be used, even if p ⩽ 0.05. In such a case one must consider the Fisher's exact; f ⩽ 0.05

As far as lymph node status is concerned, the p53 marker immunohistochemical expression was positive in 50% (14/28 cases) of the cases. Of this total, 71.4% (10 cases) did not have metastasis for lymph nodes and only 28.6% (4 cases) had metastasis. When we compared the p53 immuno-positivity with lymph node status (p = 0.023); however, the Fisher's exact was higher than 0.05 (f = 0.056), thus rending the relation statistically meaningless. The positive expression combined with the p53+Ki-67 markers was also positive in 50% of the total number of cases, but only in 28.6% (4 cases) of the cases with lymph node metastasis had p value of 0.023 for this relationship, however the Fisher's exact was higher than 0.05 (f = 0.056). Comparing the Ki-67 antigen with lymph node status was also not significant, p = 0.075, although the marker's immunohistochemical expression was positive in 92.85% (26/28 cases) from the total of cases, although it was present in 85.7% of those with lymph node metastasis.

The p53 marker's immunohistochemical expression was positive in 54.2% of male patients (13/24 cases) and 25% of the patients were females (1/4 cases); notwithstanding, this relationship (p53 versus gender) was statistically significant (p = 0.28), and when we studied the relationship of p53 immuno-positivity versus pT and histological grade, it did not yield statistical meaning in any of the cases, p = 0.114 and p = 0.075, respectively.

The Ki-67 marker was positive in 92.85% of the tumors (26/28 cases), in male patients this immuno-positivity happened in 95.83% (23/24 cases) and in 75% (3/4 cases) of female patients, however this relation was not statistically significant (p = 0.134). As to tumoral staging, the cell proliferation marker was positive in 100% (10/10 cases) of the cases in which the neoplasia did not invade adjacent structures, that is, pT1, pT2 and pT3 and it was positive in 88.9% (16/18 cases) of the cases in which the neoplasia invaded adjacent structures (pT4); however this ratio Ki-67 versus pT, was not statistically significant, p = 0.523.

The cell proliferation marker, which was positive in 92.85% of all the cases was positive in 100% of the cases classified as being of low grade (2/2 cases) and in 92.3% of the cases classified as being of high grade (24/26 cases), and when compared to the degree of histological differentiation, it did not show any significance, p = 0.578. The simultaneous expression of both markers p53 + ki-67 also did not show statistical significance when correlated with gender, pT and histological grade, p = 0.280, p = 0.114 and p = 0.075.

Tumor volume was greater than 8cm^3^ in 75% (21/28 cases) of the cases. When tumor volume was compared to lymph node metastasis, tumor volume was greater than 8cm^3^ in 92.85% (13/14 cases) of the cases, this relationship proved significant, p = 0.029. The p53 marker expressed immuno-positivity in 57.1% (12/21 cases) of tumors larger than 8cm^3^ and in only 42.9% (3/7 cases) of those smaller than 8cm^3^, the volume versus p53 relation proved to be statistically significant, p = 0.029. Thus, the p53+Ki-67 combined expression when related to tumors larger than 8cm^3^ proved to be valid, p = 0.029. However, the other variables in the study such as gender, degree of histological differentiation and tumor staging (pT) did not show statistical significance, p = 0.70, p = 0.75 and p = 0.69.

## DISCUSSION

Oral cavity and tongue carcinomas are broadly studied and discussed in the world medical literature. Recent studies have clearly shown the importance of immunohistochemistry in the evaluation of predictive values and prognosis of oral cavity and tongue epidermoid carcinomas.

This study aims at assessing the p53 and Ki-67 immunohistochemical markers and to correlate the expression of these markers with prognostic factors, already known and accepted in the medical literature and others not so much, such as tumor volume.

The immunohistochemical expression of p53 happened in 50% of the cases we studied, as it happened in the study carried out by Nakanishi et al.^11^ and Forastiere et al.9. The statistical analysis of the p53 immuno-positivity correlation showed a strong trend when associated with the presence of metastasis to the regional lymph nodes; however, without statistical significance, although p = 0.023; in these cases it was necessary to use the Fisher's exact, f = 0.056. Some authors were able to prove the statistical significance of this relation, overexpression of p53 and metastasis for lymph nodes in oral cavity carcinomas and also in neoplasias of other primary sites, such as the esophagus and the brain, for instance^13^. Authors such as Oliveira et al.^14^,^15^ showed in their studies that p53 presence is correlated to a larger number of metastasis to lymph nodes and, consequently, with a worse prognosis. According to Carlos de Vicente et al.^16^, the p53 immunoexpression had a worse prognosis in patients without metastasis to neck lymph nodes were strongly related. When p53 correlations were analyzed with other factors considered, such as gender (p = 0.28) and histological differentiation (p = 0.075) there was no statistical significance; however Kurokawa H et al.^17^ concluded that p53 overexpression on the borders of the tumor is associated with its histological grade (p < 0.05).

The Ki-67 cell proliferation marker was positive in 92.85% of the cases studied. The statistical analysis attempted to correlate the Ki-67 immunohistochemical expression with other prognostic factors related to oral cavity epidermoid carcinomas, however none of the relations established proved valid, and many papers have shown the importance of analyzing the rate of cell proliferation as a truly important prognostic factor in head and neck carcinomas. In our studies, we noticed a strong trend towards Ki-67 positive immunoexpression in tumors with neck metastasis - p = 0.075. According to Myong et al.^18^, the Ki-67 immunohistochemical expression proved to be significantly higher in patients with neck lymph node metastasis, thus representing an independent prognostic factor in the survival of patients with oral cavity epidermoid carcinomas; pathological staging (pTNM) was the most important prognostic variable and Ki-67 expression has a significant effect on the cumulative survival rate of these patients. Crossovers and statistical analyses with other factors investigated, such as gender (p = 0.134), pT (p = 0.166) were not statistically significant; however, Ki-67 overexpression on tumor borders, according to Kurokawa et al.^17^ is associated to its histological grade (p < 0.05).

The simultaneous immunoexpression of both markers, p53 and Ki-67 happened in 50% of the cases studied and the statistical analysis showed us that the co-expression of p53 and Ki-67 markers with metastasis to neck lymph nodes showed a strong trend in being valid, p = 0.023, however, in this case it was necessary to use Fisher's exact, f = 0.056, thus showing that the relationship is not statistically significant. The literature shows that the co-expression of p53 and Ki-67 takes part in the carcinogenesis of oral cavity epidermoid carcinomas, thus causing cell proliferation^19^. Some authors, such as Lavertu P et al.^20^, safely state that the co-expression of p53 and Ki-67 markers is associated with a lower time of survival free from disease, including lower time to neoplasia recurrence and/or an appearance of a second early primary site, thus reducing the general survival rate. It is also worth stressing that the negative combination of these two markers (p53 - / Ki-67) is associated to a lower recurrence incidence (p = 0.02), lower locoregional recurrence rate (p = 0.01) and lower incidence of a second primary tumor (p = 0.04), as well as longer disease-free survival (p = 0.02). Statistical analysis did not prove to be significant when the marker's co-expression was compared to gender and histological differentiation: p = 0.280 and p = 0.075, respectively.

Tumor volume calculation happened from the fact that the neoplasia is a solid and tridimensional mass, and not only a linear structure, thus a pT1 tumor (a tumor with 2.0cm or less in its largest dimension) may have a total volume of 8cm^3^ (2.0cm X 2.0cm X 2.0cm) and a pT3 tumor (a tumor with more than 4.0cm in its largest dimension) may have a volume of 4.1cm^3^ (4.1cm X 1.0cm X 1.0cm), in this case, the so-called pT1 tumor would be larger than the so-called pT3. It is possible to consider that, with the technological progress of image tests, measuring tumor size will become indispensable and routine to assess the development process and the prognosis of neoplasias and also that tumor volume calculation may change the staging process of some neoplasias or even create a new methodology to stage tumors, a new TNM. The threshold volumetric volume here established was of 8cm^3^, because the medical literature still does not have any protocol to be followed. In the present study, 25% of the tumors had volume ⩽8.0cm^3^ and 75% had volume >8.0cm^3^.

The statistical analysis, when carried out, compared tumor volume (> or < than 8.0cm^3^) with the variables in our study, which showed statistical significance when tumor volume was compared to lymph node involvement (pN+) (p = 0.029), tumor volume and p53 (p = 0.029) and tumor volume and p53+Ki-67 (p = 0.029). Some authors, such as Chew MH et al.^21^ concluded that tumor volume measure allows for a better and more accurate assessment of tumor status and will very likely become a prognostic indicator of a future staging model. Some papers related to neoplasias in sites other than the oral cavity mention the importance of the volumetric measure. Chua et al.^22^ say that tumor volume measuring represents a local control independent prognostic factor which seems to be better than the “t” staging of Ho's classification in nasopharyngeal carcinomas. Following the same line of research, Lee et al.^23^ proved that there is a significant difference in the survival of patients with the larger tumors (p = 0.044), despite the stage (p = 0.25) and/or initial pT (p = 0.30). However, authors such as Chua et al.^24^ were unable to statistically prove the relationship between tumor volume in nasopharyngeal carcinomas when compared to the presence of metastasis to regional lymph nodes and recurrence; nonetheless, it was seen that the 5 year surviving rate was substantially lower in those individuals in whom the tumor measured more than 15cm^3^.

In the present investigation, tumor volume measure did not show any statistically valid relationship when compared to other factors studied, gender, pT, Ki-67 and degree of histological differentiation.

## CONCLUSION

Results from our study showed that oral cavity and tongue carcinomas tend to have an overexpression of p53 and Ki-67 in those cases with metastasis to neck lymph nodes. As far as tumor volume is concerned, tumors larger than 8.0cm^3^ presented a higher incidence of metastasis to lymph nodes, greater p53 expression and co-expression of p53 and Ki-67 markers, suggesting that these patients have a worse prognosis.

The correlation of p53, Ki-67 and tumor volume with the other variables studied did not show statistical significance (p > 0.05).
